# Piebaldisme: une anomalie pigmentaire à reconnaitre: à propos d'un cas et revue de la littérature

**DOI:** 10.11604/pamj.2016.25.155.10499

**Published:** 2016-11-14

**Authors:** Hajar El Kouarty, Badr Sououd Benjelloun Dakhama

**Affiliations:** 1 Service des Urgences Médicales Pédiatriques, Hôpital d'Enfants Rabat, Faculté de Médecine et de Pharmacie, Université Mohammed V, Rabat, Maroc

**Keywords:** Piebaldisme, dépigmentation, mélanocyte, Piebaldism, depigmentation, melanocyte

## Abstract

Le Piebaldisme est une maladie autosomique dominante rare caractérisée par une anomalie congénitale de la pigmentation de la peau devenant parsemé de zones hypopigmentaires. Il est dû à une anomalie de développement des mélanocytes. Il atteint généralement la peau en exclusivité, par ailleurs il peut être associé à d'autres anomalies ou être confondu avec d'autres diagnostics différentiels. Nous présentons le cas d'un garçon de 5ans atteint de piebaldisme dans le cadre d'un phénotype dermatologique familial sans autres atteintes. Nous discuterons à travers ce cas la pathogénie, la clinique, les diagnostics différentiels ainsi que les modalités de prise en charge et les nouveaux essais thérapeutiques.

## Abstract (en)

Piebaldism is a rare autosomal dominant disorder characterized by an abnormal congenital skin pigmentation causing hypopigmented areas. It is due to an abnormal melanocytes development. It usually affects only the skin, but it may be associated with other anomalies or confused with other differential diagnoses. We report the case of a 5-year old boy with piebaldism having a family history of dermatologic phenotype without other alterations. We here highlight the pathogenesis, clinical manifestations, differential diagnosis as well as the management techniques and new therapeutic trials.

## Introduction

Le piebaldisme est un phénotype frappant de peau caractérisée par des plages de peau et de cheveux dépigmentés. Ce phénotype a été décrit longtemps à travers l'histoire, avec les premiers écrits chez les égyptiens, les grecs et les romains [1, 2]. Il est dû à une anomalie de développement des mélanocytes secondaire à une mutation du gène c-Kit au niveau du chromosome 4 [1, 2]. Nous rapportons le cas d'un enfant atteint du piebaldisme dans le cadre d'un phénotype familial.

## Patient et observation

**Figure 1:**
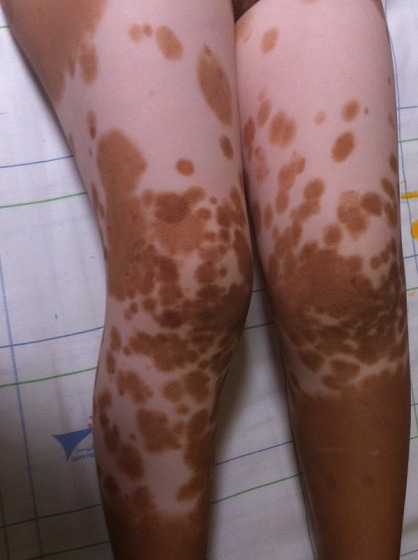
Dépigmentation symétrique de la peau avec présence de macules hypermélanotiques au sein des plaques dépigmentés

**Figure 2:**
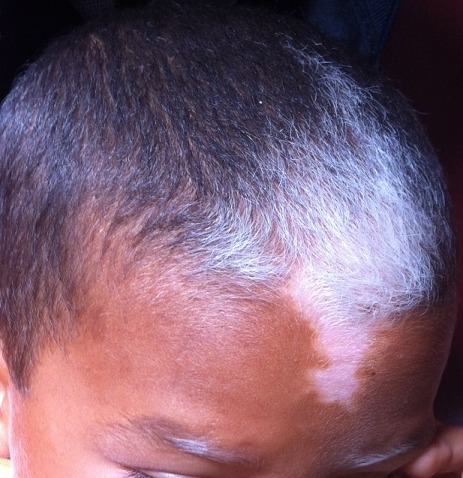
Mèche blanche caractéristique présente chez la majorité des patients atteints de piebaldisme

Il s'agit d'un enfant de 5ans, sans antécédents pathologiques particuliers, ayant consulté aux urgences pour asthénie chronique avec pâleur. L'examen clinique a retrouvé un enfant en bon état général avec des tâches achromiques diffuses intéressant le front, et de façon symétrique les avant-bras, le tronc, l'abdomen et les membres inférieurs (Figure 1). Une atteinte hypopigmentée triangulaire des cheveux en mi-cuir chevelu frontal ainsi que des tâches de dépigmentation au niveau des deux sourcils ont été décrits (Figure 2). Cette anomalie asymptomatique apparue à la naissance est également rapportée chez le père, le grand père et la tante paternelle sans association à d'autres pathologies. Le bilan biologique a retrouvé une anémie hypochrome microcytaire ferriprive pour laquelle le patient a été traité par supplémentation en fer. La recherche d'autres anomalies notamment des anomalies d'audition, de vision et des anomalies cardiovasculaires a été négative. Des conseils de protection des zones blanches de la peau ainsi que l'application d'une crème protectrice ont été prescrits pour éviter les complications. L'étude génétique a été proposée pour la famille pour déterminer la mutation responsable mais n'a pas été réalisée par faute de moyens.

## Discussion

Le piebaldisme est un phénotype très rare. Sa prévalence exacte est inconnue, mais elle a été estimée à moins de 1/20 000 naissance vivante [3, 4] La pathogénie a été reportée à une anomalie de la prolifération cellulaire et de la migration des mélanocytes pendant l'embryogenèse due à une mutation du gène c-Kit, ce dernier est responsable de la prolifération mélanocytaire [5]. Le gène c-kit a pu être localisé sur la partie proximale du bras long du chromosome 4, en 4q11-12 [6]. **Cliniquement**, Le Piebaldisme se caractérise par des plaques dépigmentées bien circonscrites de peau et de cheveux [5] présentes à la naissance, stables et persistantes, affectant la peau du visage, le tronc et les extrémités, avec une symétrie de distribution. Une mèche blanche de cheveux, souvent de forme triangulaire est présente sur la partie frontale du cuir chevelu, avec parfois atteinte du front sous-jacent [7]. 80 à 90% des patients présentent la mèche blanche uniquement [2, 5]. Les tâches dépigmentées sont généralement non progressives et persistent à l'âge adulte. Typiquement des macules hyperpigmentées supplémentaires peuvent se développer au niveau des marges ou dans les plaques blanches. Une repigmentation partielle ou complète peut se produire spontanément ou après un traumatisme chez certains patients [3, 8]. Certains enfants peuvent développer des lésions café au lait et être diagnostiqués à tort comme ayant simultanément la neurofibromatose de type I et Piebaldisme. Si la neurofibromatose de type I est suspectée, les patients doivent être soigneusement évalués pour d'autres critères de diagnostic de ce syndrome, car il peut y avoir des cas d'association avec piebaldisme [3, 9]. Il n'existe généralement pas de manifestations extracutanées, sauf dans un petit nombre de familles dans lesquelles on observe une surdité [5].

Histologiquement, les mélanocytes sont totalement absents ou très rares dans les plages de peau blanche [2] mais sont présent en quantité normale dans les zones hyperpigmentés [3]. À ce jour, plus de 60 mutations du gène KIT ont été rapportés chez l'homme atteint de Piébaldisme dont 32 mutations faux-sens, 17 suppressions, quatre insertions, sept nucléotides site d'épissage mutations, deux non-sens et une mutation chromosomique avec inversion péricentrique [2, 10]. De rares cas d'association de Piébaldisme avec d'autres maladies, y compris le mégacôlon congénital, l'anémie de Blackfan-Diamond, la glycogénose type 1a [11] ont été décrits dans la littérature . Les poils blancs peuvent être la présentation initiale de certains syndromes génétiques, y compris le syndrome de Waardenburg, la sclérose tubéreuse de bourneville et l'albinisme.ils peuvent également être acquis dans le cadre de plusieurs pathologie incluant le vitiligo, le syndrome de Vogt-Koyanagi-Harada, le syndrome Alezzandrini, la pelade et la sarcoïdose [3]. L'albinisme et le syndrome de Waardenburg restent les principaux diagnostics différentiels du piebaldisme. L'Albinisme se caractérise par une large taille de peau dépigmentée sans macules hyperpigmentées en son sein. En outre, il existe des anomalies oculaires et l'atteinte des cheveux et plus importante [7]. Le syndrome de Waardenburg est une maladie autosomique dominante caractérisée par une mèche blanche congénitale, une leucodermie avec une distribution de piebald-like, un déplacement latéral du canthus, une racine nasale hypertrophique, une hétérochromie irienne, et une perte de l'audition [3, 12].

**Le diagnostic** Du piebaldisme reste clinique après élimination des autres diagnostics différentiels. Une confirmation par étude génétique peut être envisagée surtout dans le cadre du conseil génétique [5].

**Le traitement** Repose sur la nécessité de protection des plaques blanches pauvres en mélanocytes contre les coups de soleils, ainsi que l'application de crèmes protectrices pour prévenir la transformation maligne [3, 5]. La peau dépigmentée dans le Piebaldisme est généralement insensible aux traitements médicamenteux et à la photothérapie [3]. Le maquillage de camouflage peut être proposé mais n'est qu'une solution momentanée [3, 5]. La pigmentation artificielle par le dihydroxyacétone (produit utilisé pour bronzage sans soleil) a été décrite mais constitue également une solution temporaire [3, 13]. Des essais thérapeutiques chirurgicaux ont été rapportés avec des succès variables [3], parmi eux, la transplantation autologue d'un nombre de mélanocytes qui peut atteindre 100% de repigmentation, elle consiste en un transfert de mélanocytes par greffe totale ou partielle de la peau, minigrafting ou bulles d'aspiration. Les mélanocytes peuvent également être obtenus par culture des mélanocytes ou des mélanocytes-kératocytes [14]. La transplantation micropunch (Minigrafting) épidermique totale en utilisant des sites de 1 à 1,25 mm donatrices épidermiques est une méthode relativement peu coûteuse et efficace, mais elle est limitée par les cicatrices du site donneur [3, 14].

## Conclusion

Le piebaldisme est considéré comme un phénotype relativement bénin, mais peut être socialement et psychiquement invalidant surtout chez les enfants, ceci présente un défi thérapeutique et doit mener à des recherches thérapeutiques plus approfondies incluant la thérapie génique pour améliorer la qualité de vie des patients.
